# Association of leukocyte mitochondrial DNA copy number with longitudinal C-reactive protein levels and survival in older adults: a cohort study

**DOI:** 10.1186/s12979-022-00322-8

**Published:** 2022-12-09

**Authors:** I-Chien Wu, Chin-San Liu, Wen-Ling Cheng, Ta-Tsung Lin, Hui-Ling Chen, Pei-Fen Chen, Ray-Chin Wu, Chen-Wei Huang, Chao A. Hsiung, Chih-Cheng Hsu

**Affiliations:** 1 Institute of Population Health Sciences, National Health Research Institutes, 35 Keyan Road, Zhunan, Miaoli County 35053 Taiwan; 2 Vascular and Genomic Center, Institute of ATP, Changhua Christian Hospital, Changhua, Taiwan; 3 Department of Neurology, Changhua Christian Hospital, Changhua, Taiwan; 4 Graduate Institute of Integrated Medicine, College of Chinese Medicine, China Medical University, Taichung, Taiwan; 5 Department of Post-Baccalaureate Medicine, College of Medicine, National Chung Hsing University, Taichung, Taiwan

**Keywords:** Aging, Inflammation, Mitochondria, Human, Epidemiology

## Abstract

**Background:**

Systemic chronic inflammation occurs with age. The association of the leukocyte mitochondrial DNA copy number, a measure of mitochondrial function in aging, with the temporal profile of serum high-sensitivity C-reactive protein and mortality risk remains uncertain. The objectives of this study were to examine the association of the leukocyte mitochondrial DNA copy number with longitudinal high-sensitivity C-reactive protein levels and the association of the longitudinal high-sensitivity C-reactive protein levels with mortality risk.

**Methods:**

This prospective cohort study included 3928 adults aged ≥ 55 years without systemic inflammation in the baseline examination of the Healthy Aging Longitudinal Study in Taiwan, which started in 2009. Each participant received leukocyte mitochondrial DNA copy number measurement using a fluorescence-based quantitative polymerase chain reaction at baseline, serum high-sensitivity C-reactive protein measurements at baseline and the follow-up examination five years later, and the ascertainment of all-cause death (until November 30, 2021). The relationships among the leukocyte mitochondrial DNA copy number, longitudinal serum high-sensitivity C-reactive protein levels, and time to all-cause mortality were examined using the joint longitudinal and survival modeling analysis.

**Results:**

Of the 3928 participants (mean age: 69 years; 2060 [52%] were women), 837 (21%) died during follow-up. In the adjusted analysis, one standard deviation lower natural log-transformed baseline leukocyte mitochondrial DNA copy number was associated with an increase of 0.05 (95% confidence interval [CI], 0.02 to 0.08) standard deviation in serum high-sensitivity C-reactive protein in subsequent years. An increase of 1 standard deviation in instantaneous high-sensitivity C-reactive protein levels was associated with a hazard ratio (HR) for all-cause mortality of 1.22 (95% CI, 1.14 to 1.30). Similar results were obtained after further adjusting for baseline high-sensitivity C-reactive protein levels (HR [95% CI], 1.27 [1.16 to 1.38]) and after excluding those with serum high-sensitivity C-reactive protein above 10 mg/L (HR [95% CI], 1.21[1.11 to 1.31]) or 3 mg/L (HR [95% CI], 1.19 [1.06 to 1.31]) during follow-up.

**Conclusions:**

A lower leukocyte mitochondrial DNA copy number was associated with persistently higher high-sensitivity C-reactive protein levels. Moreover, these higher time-varying high-sensitivity C-reactive protein levels were instantaneously associated with a higher risk of death.

**Supplementary Information:**

The online version contains supplementary material available at 10.1186/s12979-022-00322-8.

## Introduction

Chronically high blood levels of C-reactive protein (CRP), an acute-phase protein induced by the systemic effects of proinflammatory cytokines [1], and some proinflammatory cytokines themselves are frequently observed in older individuals, even those who appear healthy [2, 3]. Of note, multiple lines of evidence have indicated that simply having a higher blood level of CRP is associated with greater risks of age-related chronic diseases, chronic health conditions, and multiple adverse health outcomes [2–6]. As the population ages, the prevalence of these inflammaging-related health problems, which is already sizable, is likely to increase.

However, the systemic chronic inflammation that tends to develop with age remains incompletely understood [2, 3]. The systemic chronic inflammation has been theorized as a key feature of the aging process [3, 7–9]. Aging is associated with alterations in mitochondria [9]. The mitochondrion has its own genome, which encodes components of the oxidative phosphorylation system and exists in multiple copies [10, 11]. The mitochondrial DNA content of peripheral blood cells, usually measured as the quantity of mitochondrial DNA relative to that of nuclear DNA (mitochondrial DNA copy number [mtDNA_CN_]) [9, 11–14], often decreases with age and is a potential biomarker of mitochondrial function in aging [15–18]. Studies have demonstrated that the lower the mtDNA_CN_ of blood cells is, the greater the risk of specific age-related diseases is [12, 19–21]. These diseases include, but not limited to, coronary heart disease, stroke, kidney function decline, and neurodegenerative disorders. For instance, Ashar et al. [19] showed that one standard deviation lower leukocytes mtDNA_CN_ was associated with greater risks of incident coronary heart disease and stroke. Tin et al. [20] demonstrated that blood cells mtDNA_CN_ at the highest quartile was associated with a lower risk of incident chronic kidney disease. These findings suggest that blood cells mtDNA_CN_ is inversely related to the risk of these diseases. Notably, chronic inflammation is a common feature of many of these diseases, in particular coronary heart disease, ischemic stroke, and kidney function decline [5, 22]. Therefore, an inference from these observations is that a lower blood cell mtDNA_CN_ may be related to systemic chronic inflammation. In line with this, accumulating experimental evidence suggests that immune cell mitochondria are active players in immunity and inflammation and may play etiological roles in the inflammatory pathogenic process [23, 24].

The blood levels of high-sensitivity CRP (hs-CRP) are primarily determined by its synthesis rate in hepatocytes, which in turn is determined by the levels of circulating proinflammatory cytokines raised by a distal inflammatory pathogenic process [1]. As such, serum hs-CRP is a commonly used clinical laboratory test, and its high level typically indicates an active inflammatory pathogenic process. Most studies on hs-CRP have focused on levels measured at a single time point. Although its blood levels at a single time point carry rich prognostic information [2], more recent studies suggested that its temporal profile may reflect the activity of the corresponding inflammatory pathogenic process in a more timely and proportional manner and that it may additionally provide more detailed information about the activity of the process [25, 26].

The aim of this study was twofold (Additional file 1: Supplementary Fig. 1). The first aim was to prospectively evaluate the relationship of the peripheral blood leukocyte mtDNA_CN_ with longitudinal hs-CRP levels in a large cohort of older persons without systemic inflammation. The second aim was to assess the relationship between the longitudinal hs-CRP levels and mortality risk. We hypothesized that a lower peripheral blood leukocyte mtDNA_CN_ would be associated with persistently higher time-varying hs-CRP levels and that these higher time-varying hs-CRP levels would be associated with a higher risk of death after adjusting for the effects of multiple potential confounders.

## Methods

### Study design and participants

We analyzed prospectively collected data from the Healthy Aging Longitudinal Study in Taiwan (HALST). The details of the HALST were published previously [27]. In brief, the HALST is an ongoing prospective population-based cohort study in Taiwan that started in 2009 with 5663 community-dwelling adults aged 55 years and older enrolled. After being recruited, these participants received a baseline (first wave) examination and a follow-up (second wave) examination five years later. Sociodemographic status, lifestyles, diseases, and health status were assessed during these examinations. In addition, venous blood samples were collected, immediately processed, and maintained at -80 °C until assayed. This study was approved by the institutional review boards of the National Health Research Institutes and participating hospitals.

This study examined HALST participants without systemic inflammation, defined as a serum hs-CRP level of 2 mg per liter or higher [25, 28, 29], at baseline examination, leaving 3928 HALST participants entering this study. Supplementary Table 1 (of Additional file 1) showed the characteristics of the excluded participants. Each participant received measurement of leukocyte mtDNA_CN_ at baseline and measurements of serum hs-CRP at baseline and the follow-up examination.

### Measurement of peripheral blood leukocyte mitochondrial DNA copy number

The leukocyte mtDNA_CN_ was measured in accordance with protocols described in previous studies [30]. In brief, we amplified a mitochondrial gene (ND1 gene) and a nuclear gene (β-globin gene) in the leukocyte total cellular DNA extracted from venous blood samples using a fluorescence-based quantitative polymerase chain reaction. The relative mtDNA_CN_ was estimated on the basis of these two genes’ threshold cycle numbers.

### Measurement of serum high-sensitivity C-reactive protein

Baseline and follow-up serum hs-CRP levels were measured using a latex-enhanced immunoturbidimetric assay (ADVIA 1800 Chemistry system, Siemens AG, Munich, Germany). The intra-assay coefficient of variation was 5.61%, and the lowest detectable concentration was 0.12 mg/L.

### Assessment of mortality

By linking to data from the Bureau of National Health Insurance of Taiwan death registry, any death events that occurred during the follow-up period were identified systematically, and the date of each death was ascertained. We followed each participant from the date of baseline examination (index date) until death or November 30, 2021, whichever occurred first.

### Measurement of covariate variables

Other variables were measured during the baseline examinations. These variables were age (years), sex, education level (illiteracy, elementary school, junior high school, ≥ high school), smoking status (current, former, never), body mass index (BMI), dyslipidemia (serum triglycerides, serum high-density lipoprotein [HDL] cholesterol, serum low-density lipoprotein [LDL] cholesterol), and comorbidities (hypertension, cardiometabolic diseases [diabetes mellitus, stroke, cardiovascular disease], chronic kidney disease, lung disease). Further details are presented in the Supplementary Materials (Additional file 1: Supplementary Methods).

### Statistical analyses

The baseline characteristics of the participants were summarized using descriptive statistics (mean [standard deviation] for normally distributed continuous variables, median [interquartile range] for nonnormally distributed continuous variables, and numbers [percentage] for categorical variables). Because both hs-CRP and the mtDNA_CN_ have a skewed distribution, both were analyzed as natural log-transformed values throughout the remainder of the analysis.

We jointly modeled the association of the leukocyte mtDNA_CN_ with longitudinal serum hs-CRP levels and time to all-cause mortality using maximum likelihood [31, 32]. The modeling details are presented in the Supplementary Materials (Additional file 1: Supplementary Methods). In the longitudinal part of the joint analysis, we modeled serum hs-CRP levels over time using linear mixed models (random-intercept-and-random-slope models). Each model incorporated fixed effects for the leukocyte mtDNA_CN_, time (in years) since baseline examination, time’s interactions with the leukocyte mtDNA_CN_, and random effects for intercept and slope with an unstructured variance–covariance structure. Departure from linearity was detected using restricted cubic splines with three knots. The correlations of longitudinal data for each individual were accommodated by the random effects in these models. We examined the directions and magnitudes of associations of the baseline leukocyte mtDNA_CN_ with hs-CRP based on parameter estimates.

In the survival part of the joint analysis, we modeled the association of the instantaneous hs-CRP level at time t (current value parameterization), as estimated by the longitudinal sub-models, with the risk of death at that time by using a Weibull parametric survival model. Based on the parameter estimates in the model, the hazard ratio (HR) per 1 standard deviation (SD) increase in log-transformed hs-CRP was estimated and presented.

**Fig. 1:**
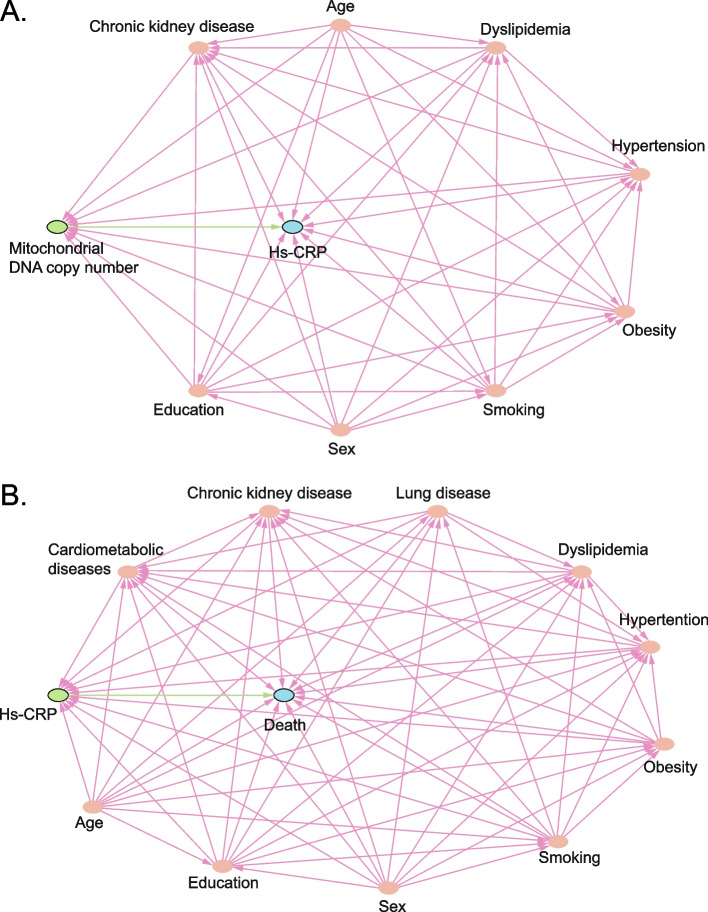
Directed acyclic graph depicting the assumed relationship among leukocyte mitochondrial DNA copy number, serum high-sensitive C-reactive protein, risk of death, and the potential confounders. Panel **A** displayed the relationships among leukocyte mitochondrial DNA copy number (exposure, represented by the green circle), serum high-sensitive C-reactive protein (outcome, represented by the blue circle), and covariates associated with both the leukocyte mitochondrial DNA copy number and serum high-sensitive C-reactive protein (ancestors of both the exposure and outcome, represented by the red circles). Panel **B** displayed the relationships among serum high-sensitive C-reactive protein (exposure, represented by the green circle), risk of death (outcome, represented by the blue circle), and the covariates associated with both serum high-sensitive C-reactive protein and risk of death (ancestors of both the exposure and outcome, represented by the red circles). The arrow from variable X to variable Y represents a direct effect of X on Y (an effect not mediated by other observed variables in the graph) that may have been present. The hypothesized causal pathway is presented in green. The biasing pathway is presented in red [34]

Adjustments were made for covariates in the longitudinal (fixed effects for covariates and their interactions with time) and survival sub-models of joint analysis. As suggested in the literature, we identified a priori covariates that could act as potential confounders and a minimally sufficient set of covariates for adjustment by using a directed acyclic graph (DAG; Fig. 1) [33, 34]. In the longitudinal sub-model, the set of covariates was age, sex, educational level, smoking status, obesity (BMI), dyslipidemia, hypertension, and chronic kidney disease. In the survival sub-model, the set of covariates was age, sex, educational level, smoking status, obesity (BMI), dyslipidemia, hypertension, cardiometabolic diseases, lung disease, and chronic kidney disease. The joint models’ assumptions were examined using residual plots. The proportional hazards assumption of the survival part was evaluated using log–log survival curves and observed-versus-predicted survival curves.

We conducted several sensitivity analyses. First, we added the baseline value of hs-CRP in the survival part of the joint analysis. Second, in the survival part of the joint analysis, we assumed that the risk of death at time *t* depends on the instantaneous hs-CRP level at that time. However, the risk of death at time *t* may depend on the levels of hs-CRP prior to time *t*. To examine whether the assumption might affect the results, we additionally modeled the association of the area under the hs-CRP trajectory curve (in mg/L × years) up to time *t* (cumulative effects parameterization), which captured the effects of the whole inflammation exposure history up to time *t*, with the risk of death at time *t*. Third, to minimize the effects of infectious processes, we excluded all participants with serum hs-CRP above 10 mg/L during follow-up (*n* = 82) and then repeated the analysis [35]. We then repeated the analysis again after excluding those with serum hs-CRP above 3 mg/L during follow-up (*n* = 319). Fourth, we conducted a sensitivity analysis to evaluate the robustness of our observations against potential unmeasured confounders by calculating the E-value [36]. E-value quantifies the minimal strength (on the risk-ratio scale) of the association of unmeasured confounders with both exposure and outcomes, conditional on the measured confounders, that can fully explain the observed exposure-outcome association.

Statistical significance was defined as a two-sided *P* value lower than 0.05. Analyses were performed using STATA, version 16.1 (StataCorp LP., College Station, TX, USA) and R, versions 4.1.2 (R Foundation for Statistical Computing).

## Results

The characteristics of the study participants at baseline are presented in Table 1. The mean age was 69 years, and 52% of the participants were women. The median leukocyte mtDNA_CN_ at baseline was 72.00. Of the 3928 participants, 837 died during a median follow-up period of 10.6 years. Participants who died during the follow-up period had a significantly lower leukocyte mtDNA_CN_ at baseline (68.00) than those who survived (73.00).

**Table 1:** Baseline characteristics of study participants^a^

	**All participants**	**Participants who survived the follow-up**	**Participants who died during the follow-up**	
**Characteristics**	(*n* = 3,928)	(*n* = 3,091)	(*n* = 837)	***P***^b^
Leukocyte mitochondrial DNA copy number, median (interquartile range)	72.00 (59.00–87.25)	73.00 (60.00–88.50)	68.00 (56.00–83.50)	.0094
High-sensitivity C-reactive protein, median (interquartile range), mg/L	0.39 (0.16–0.83)	0.38 (0.16–0.80)	0.46 (0.17–0.94)	< .0001
Age, years	69.1 (8.1)	67.4 (7.3)	75.4 (7.9)	< .0001
Women, n (%)	2060 (52.4)	1728 (55.9)	332 (39.7)	< .0001
Education				
Illiteracy, n (%)	370 (9.4)	243 (7.9)	127 (15.2)	< .0001
Elementary school, n (%)	1667 (42.4)	1264 (40.9)	403 (48.2)	
Junior high school	447 (11.4)	355 (11.5)	92 (11.0)	
≥ High school, n (%)	1444 (36.8)	1229 (39.8)	215 (25.7)	
Smoking				
Never, n (%)	2830 (72.1)	2337 (75.6)	493 (58.9)	< .0001
Former smoker, n (%)	634 (16.1)	434 (14.0)	200 (23.9)	
Current smoker, n (%)	464 (11.8)	320 (10.4)	144 (17.2)	
Body mass index, kg/m^2^	24.2 (3.3)	24.3 (3.3)	24 (3.6)	.0089
Serum triglycerides, mg/dL	119.1 (78.8)	120 (81.4)	115.9 (68.4)	.1822
Serum high-density lipoprotein cholesterol, mg/dL	53.5 (13.9)	53.9 (13.7)	52.2 (14.4)	.0013
Serum low-density lipoprotein cholesterol, mg/dL	116.6 (32.0)	118.2 (31.4)	110.4 (33.4)	< .0001
Hypertension, n (%)	1988 (50.6)	1443 (46.7)	545 (65.1)	< .0001
Diabetes mellitus, n (%)	1021 (26.0)	708 (22.9)	313 (37.4)	< .0001
Cardiovascular disease, n (%)	799 (20.3)	577 (18.7)	222 (26.5)	< .0001
Stroke, n (%)	188 (4.8)	100 (3.2)	88 (10.5)	< .0001
Lung disease, n (%)	116 (3.0)	72 (2.3)	44 (5.3)	< .0001
Chronic kidney disease, n (%)	559 (14.2)	295 (9.5)	264 (31.5)	< .0001
Years of observation, years, median (interquartile range)	10.6 (9.1–11.6)	11.1 (9.6–12.0)	7.0 (4.7–9.1)	< .0001

^a^Data are mean (standard deviations) unless otherwise specified

^b^Continuous variables were analyzed using a one-way analysis of variance, whereas categorical variables (proportions) were analyzed using the chi-square test

### Relationship of peripheral blood leukocyte MtDNA_CN_ with longitudinal hs-CRP levels

The longitudinal part of the joint analysis revealed that among the participants without systemic inflammation at baseline, those with a lower baseline leukocyte mtDNA_CN_ were more likely to have higher serum hs-CRP levels in subsequent years (Table 2). After adjustment, the baseline leukocyte mtDNA_CN_ remained inversely associated with serum hs-CRP. A 1-SD lower natural log-transformed baseline leukocyte mtDNA_CN_ was associated with an increase of 0.05-SD in serum hs-CRP levels throughout the subsequent years. The participants with a baseline leukocyte mtDNA_CN_ within the lowest tertile (< 63.5) had 0.11-SD (95% CI, 0.04 to 0.18) increased serum hs-CRP levels compared with those with a baseline leukocyte mtDNA_CN_ within the highest tertile (> 81). We did not observe a non-linear relationship.

**Table 2:** Association of baseline leukocyte mitochondrial DNA copy number with high-sensitivity C-reactive protein trajectory^a^

**Model**		**Model 1**			**Model 2**	
**Variable**	**Effects**^b^	**95% CI**	***P***	**Effects**^b^	**95% CI**	***P***
Intercept	0.01	-0.02 to 0.04	.688	-2.44	-2.88 to -2.00	< .001
Mitochondrial DNA copy number	-0.06	-0.09 to -0.03	< .001	-0.05	-0.08 to -0.02	.002
Age				0.01	0.01 to 0.01	< .001
Sex						
Women					(Reference)	
Men				-0.25	-.033 to -0.18	< .001
Smoking						
Never					(Reference)	
Former smoker				0.17	0.07 to 0.27	< .001
Current smoker				0.28	0.17 to 0.38	< .001
BMI				0.06	0.05 to 0.07	< .001
Serum triglycerides				0.001	0.001 to 0.002	< .001
Serum high-density lipoprotein cholesterol				-0.004	-0.007 to -0.002	< .001
Serum low-density lipoprotein cholesterol				0.004	0.003 to 0.005	< .001
Hypertension				0.02	-0.04 to 0.09	.74
Chronic kidney disease				0.09	-0.001 to 0.181	.052
Time	0.08	0.07 to 0.09	< .001	0.03	0.01 to 0.05	< .001
Mitochondrial DNA copy number x time	-0.0001	-0.0091 to 0.0090	.990	Not included		
Sex x time						
Women					(Reference)	
Men				0.03	0.01 to 0.05	.001
Education x time						
Illiteracy				0.08	0.05 to 0.12	< .001
Elementary school				0.04	0.02 to 0.06	< .001
Junior high school				0.04	0.01 to 0.07	.011
≥ High school					(Reference)	
Chronic kidney disease x time				0.05	0.02 to 0.08	.001

^a^The results of the longitudinal part of the joint analysis, in which the association between the baseline leukocyte mitochondrial DNA copy number and the serum high-sensitivity C-reactive protein levels (both of which were natural log-transformed and standardized) was modeled using linear mixed regression with adjustment for other model variables

^b^Change in high-sensitivity C-reactive protein levels (in 1 standard deviation)

We observed similar results when we excluded the participants with serum hs-CRP above 10 mg/L during follow-up (Additional file 1: Supplementary Table 2). A 1-SD lower natural log-transformed baseline leukocyte mtDNA_CN_ was associated with an increase of 0.05-SD in serum hs-CRP levels throughout the subsequent years. After the further exclusion of all participants with serum hs-CRP above 3 mg/L during follow-up, a 1-SD lower natural log-transformed baseline leukocyte mtDNA_CN_ was associated with an increase of 0.04-SD in serum hs-CRP levels (Additional file 1: Supplementary Table 3).

### Relationship between the longitudinal hs-CRP levels and mortality risk

Next, we examined whether the trajectory of serum hs-CRP during follow-up was associated with mortality risk (Table 3). The survival part of the joint analysis revealed that a 1-SD increase in instantaneous hs-CRP levels was associated with an HR for all-cause mortality of 1.36 (95% CI, 1.27 to 1.46) in the unadjusted model and 1.22 (95% CI, 1.14 to 1.30) in the fully adjusted model.

**Table 3:** Association between estimated high-sensitivity C-reactive protein trajectory and risk of mortality^a^

	**All**			**Excluding participants (*n* = 82) with hs-CRP > 10 mg/L during follow-up**			**Excluding participants (*n* = 319) with hs-CRP > 3 mg/L during follow-up**		
**Trajectory feature**	**Hazard ratio**^b^	**95% CI**	***P***	**Hazard ratio**^b^	**95% CI**	***P***	**Hazard ratio**^b^	**95% CI**	***P***
Instantaneous hs-CRP level (mg/L)									
Unadjusted	1.36	1.27 to 1.46	< .001	1.37	1.25 to 1.48	< .001	1.37	1.20 to 1.54	< .001
Multivariable adjusted^c^	1.22	1.14 to 1.30	< .001	1.21	1.11 to 1.31	< .001	1.19	1.06 to 1.31	< .001

^a^Hs-CRP, high-sensitivity C-reactive protein

^b^Hazard ratio per 1 standard deviation increase in hs-CRP levels

^c^Adjusted for age, sex, educational level, smoking status, obesity (BMI), dyslipidemia (serum triglycerides, HDL cholesterol, LDL cholesterol), hypertension, cardiometabolic diseases (diabetes mellitus, stroke, cardiovascular disease), lung disease, and chronic kidney disease

When the baseline value of hs-CRP was added in the survival part of the joint analysis, we observed an HR for all-cause mortality of 1.27 (95% CI, 1.16 to 1.38) associated with a 1-SD increase in instantaneous hs-CRP levels. The term for baseline hs-CRP did not reach statistical significance. We likewise observed an association between the entire hs-CRP exposure during the follow-up period (mg/L × years) and the risk of death (Additional file 1: Supplementary Table 4). One standard deviation increase in hs-CRP levels × years was associated with an HR for all-cause mortality of 1.05 (95% CI, 1.04 to 1.07) in the unadjusted model and 1.03 (95% CI, 1.02 to 1.04) in the fully adjusted models. We also examined whether the estimated effects of hs-CRP on mortality risk were explained by hs-CRP levels exceeding clinically relevant thresholds. In general, the analysis yielded similar results after we excluded the participants with serum hs-CRP above 10 mg/L during follow-up and even after the further exclusion of those with serum hs-CRP above 3 mg/L during follow-up (Table 3) (Additional file 1: Supplementary Table 4). The E-values on the risk ratio scales for the unadjusted and adjusted HR estimates and their lower confidence limit are presented in Supplementary Tables 5, 6, and 7 of additional file 1.

## Discussion

In this longitudinal study of community-dwelling older adults, we identified the relationship of peripheral blood leukocyte mtDNA_CN_ with longitudinal serum hs-CRP levels and the relationship of the longitudinal serum hs-CRP levels with the risk of death. This study was the first to demonstrate that even without accompanying signs of systemic inflammation, a lower peripheral blood leukocyte mtDNA_CN_ at baseline was associated with persistently higher hs-CRP levels. Moreover, our analysis indicated that these higher longitudinal hs-CRP levels were associated with a higher risk of death. These associations were independent of baseline hs-CRP levels, infectious processes, high-risk cardiovascular inflammation and potential confounders.

Blood levels of hs-CRP are a well-recognized and extensively used biomarker of inflammation [1, 25, 26, 35, 37, 38]. As an acute-phase protein, CRP is rapidly synthesized in hepatocytes, stimulated mainly by circulating cytokine interleukin-6, and released into circulation in response to a range of inflammatory pathologies at distant sites in a dose–response manner [1, 39, 40]. With a constant half-life in the circulation, the level of hs-CRP in an individual’s blood is mainly determined by its rate of synthesis, which in turn is determined by the activity of the underlying cytokines-producing inflammatory pathologies [41]. Therefore, an examination of an individual’s temporal profile of serum hs-CRP by measuring the levels of hs-CRP in their blood serially—rather than at a single time point, as in most studies—offers a unique opportunity to reliably track the activity of the underlying inflammatory pathogenic process [1, 25, 26, 37, 38].

To the best of our knowledge, this study is the first to examine the relationship between the peripheral blood leukocyte mtDNA_CN_ and the temporal profile of serum hs-CRP. We demonstrated that among older adults free of clinically relevant systemic inflammation, a lower peripheral blood leukocyte mtDNA_CN_ was associated with a persistently higher level of hs-CRP in the subsequent years. This observation was consistent across the multiple analyses that took into account the potential effects of confounders and intercurrent hs-CRP-raising disease processes. These results suggest that a lower leukocyte mtDNA_CN_ may be associated with an active inflammatory pathogenic process throughout the years. In general, the observation of an inverse relationship between the peripheral blood leukocyte mtDNA_CN_ and serum hs-CRP is consistent with the results of other studies, although most analyses have been cross-sectional in nature and thus have precluded the determination of the chronicity of hs-CRP elevation. For instance, in another study, we revealed that the peripheral blood leukocyte mtDNA_CN_ was negatively correlated with hs-CRP in a cohort of 1990 older adults [42]. In a cohort of 9058 participants without chronic kidney disease [20], Tin et al. noted that those with lower peripheral blood cells mtDNA_CN_ had a higher serum hs-CRP level. In another cohort of 4812 patients with chronic kidney disease [43], those with a lower whole blood mtDNA_CN_ were also observed to have a higher hs-CRP.

In this study, by conducting the joint longitudinal and survival analysis, we further demonstrated that a higher level of hs-CRP during follow-up was instantaneously associated with a higher risk of death independent of confounders. Most importantly, this effect of follow-up hs-CRP levels on death risk was independent of baseline hs-CRP levels. On the other hand, the baseline hs-CRP levels were not associated with the death risk after adjusting for the longitudinal hs-CRP levels. Interestingly, after excluding individuals with follow-up hs-CRP levels exceeding thresholds that may indicate an intercurrent infection or high-risk cardiovascular inflammation, the study sample consisted of older adults likely at average or lower risk of diseases (e.g., cardiovascular diseases). Nevertheless, the effects were still observed in this sample of older adults. In sensitivity analysis, we examined whether a different analytic assumption about how the death risk depends on the longitudinal hs-CRP levels might affect the results. Assuming that the risk of death at time *t* depends on the whole hs-CRP exposure history up to time *t*, we still observed significant, albeit small, effects of hs-CRP level on the risk of death. The overall results of the survival analysis agree with the argued values of serially determining hs-CRP levels and imply that the longitudinal hs-CRP levels may carry additional information about the near-term health event hazard. Even below clinically relevant thresholds, the chronic low-grade inflammatory process associated with a lower leukocyte mtDNA_CN_ might uniquely confer an immediately higher mortality risk in older adults, including low-risk individuals. However, we could not exclude the possibility that the chronic low-grade inflammatory process caused by factors other than lower leukocyte mtDNA_CN_ confers a higher mortality risk.

The findings of this longitudinal study are consistent with recent results of experimental investigations that have suggested a causal role of blood cell mitochondria in the development of pathogenic systemic chronic inflammation. First, emerging evidence from human studies has indicated that a lower blood cells mtDNA_CN_ may reflect a lower expression of many mitochondrial- and nuclear-encoded genes involved in mitochondrial DNA replication and function in blood cells [13]. Second, as the hub of multiple metabolic and signaling pathways, mitochondria in immune cells play pivotal roles in shaping immune and inflammatory responses [23]. Third, experiments in mice have demonstrated that decreased activity in immune cells' mitochondria metabolic pathways, including mitochondrial respiration, drives systemic chronic inflammation in aging, thereby, the development of age-related diseases [24].

The major strengths of this study include its prospective design with serial measurements of hs-CRP and the use of an advanced analytical method suitable for studying events with a mechanism of significant chronicity (e.g., aging). In addition, this study analyzed prospectively collected data from a well-established cohort with comprehensive baseline information obtained through a standardized procedure. Notably, the measurements of the mtDNA_CN_ and hs-CRP in this study were centralized and standardized. All these characteristics helped this study minimize the likelihood of observing spurious associations. Nevertheless, our study has some limitations. First, as an observational study, inferences regarding causality and mechanism should be drawn with caution. Second, although we identified potential confounders a priori and, guided by DAGs, attempted to adjust for confounding bias during model building, residual confounding by unmeasured or uncontrolled covariates still could not be excluded. We could not adjust for the potential confounding effects of time-varying covariates on the association between higher longitudinal hs-CRP levels and a higher risk of death. To assess how likely the observed association could be due to uncontrolled confounding, we calculated the E-value in the sensitivity analysis. We found that an uncontrolled covariate would need to be associated with both the time-varying hs-CRP levels and mortality risk with a risk ratio of at least 1.56 (lower limit: 1.42) to explain the observed association fully. Baseline stroke was the covariate most strongly associated with mortality, with an HR of 1.74. After excluding stroke from the model, we still obtained an E-value of 1.56 with a lower confidence limit of 1.42. We reasoned that the observed association was unlikely to be due to uncontrolled confounding. Third, the sample of this observational study consisted of adults aged 55 years and older at study entry. We could not completely exclude the possibility of survival bias and the possibility of underestimating the effects of lower leukocyte mtDNA_CN_. Fourth, we excluded those with higher baseline hs-CRP from this study to examine the association of leukocyte mtDNA_CN_ with hs-CRP trajectory among those who had not yet developed systemic inflammation at the time of mtDNA_CN_ measurement. As such, our findings may not be generalizable to other populations of older adults who might be more ill. Fifth, further research would be worthwhile to determine the clinical relevance of the sub-threshold hs-CRP levels in older adults. Sixth, we showed that the inflammatory process might have a cumulative effect on the risk of death, as demonstrated by the sensitivity analysis. The clinical relevance of such findings is unclear and is needed to be further determined. Finally, both high and low blood cells mtDNA_CN_ have been associated with higher risks of a variety of cancers. The roles of inflammation in this context would also be worth investigating.

## Conclusions

In conclusion, among older persons without systemic inflammation at baseline, a lower peripheral blood leukocyte mtDNA_CN_ was associated with persistently higher hs-CRP levels. Moreover, the higher time-varying hs-CRP levels were immediately associated with higher risks of death. Further studies are warranted to clarify the underlying mechanisms.

## Supplementary Information

**Additional file 1: Supplementary Figure 1.** The assumed relationship among leukocyte mitochondrial DNA copy number, serum high-sensitive C-reactive protein, risk of death. **Supplementary Methods.** Measurement of Covariate Variables, Statistical Analyses, References. **Supplementary Table 1.** Characteristics of HALST Participants Excluded from This Study. **Supplementary Table 2.** Association of Baseline Leukocyte Mitochondrial DNA Copy Number with High-Sensitivity C-Reactive Protein Trajectory After Excluding Participants with High-Sensitivity C-Reactive Protein >10 mg/L During Follow-Up. **Supplementary Table 3.** Association of Baseline Leukocyte Mitochondrial DNA Copy Number with High-Sensitivity C-Reactive Protein Trajectory After Excluding Participants with High-Sensitivity C-Reactive Protein >3 mg/L During Follow-Up. **Supplementary Table 4.** Association between Estimated High-Sensitivity C-Reactive Protein Trajectory and Risk of Mortality. **Supplementary Table 5.** E-Values for the Association of Features of Estimated High-Sensitivity C-Reactive Protein Trajectory Over Time With Risk of Mortality in All Participants. **Supplementary Table 6.** E-Values for the Association of Features of Estimated High-Sensitivity C-Reactive Protein Trajectory Over Time With Risk of Mortality after the Exclusion of Participants With High-Sensitivity C-Reactive Protein > 10 mg/L During Follow-Up. **Supplementary Table 7.** E-Values for the Association of Features of Estimated High-Sensitivity C-Reactive Protein Trajectory Over Time With Risk of Mortality after the Exclusion of Participants With High-Sensitivity C-Reactive Protein > 3 mg/L During Follow-Up.

## Data Availability

The dataset supporting the conclusions of this article is not publicly available according to the Personal Information Protection Act of Taiwan and the regulations of the National Health Research Institutes.

## Abbreviations

BMI: Body mass index
CRP: C-reactive protein
DAG: Directed acyclic graph
HALST: Healthy Aging Longitudinal Study in Taiwan
HDL cholesterol: High-density lipoprotein cholesterol
hs-CRP: High-sensitivity CRP
LDL cholesterol: Low-density lipoprotein cholesterol
mtDNA_CN_: Mitochondrial DNA copy number
