# Supplementary material for: Association of leukocyte mitochondrial DNA copy number with longitudinal C-reactive protein levels and survival in older adults: a cohort study
Source: Immun Ageing. 2022 Dec 9;19:62. doi: 10.1186/s12979-022-00322-8 (PMC9733307; doi:10.1186/s12979-022-00322-8)
Supplement: Supplementary file 1 — Additional file 1: Supplementary Figure 1. The assumed relationship among leukocyte mitochondrial DNA copy number, serum high-sensitive C-reactive protein, risk of death. Supplementary Methods. Measurement of Covariate Variables, Statistical Analyses, References. Supplementary Table 1. Characteristics of HALST Participants Excluded from This Study. Supplementary Table 2. Association of Baseline Leukocyte Mitochondrial DNA Copy Number with High-Sensitivity C-Reactive Protein Trajectory After Excluding Participants with High-Sensitivity C-Reactive Protein >10 mg/L During Follow-Up. Supplementary Table 3. Association of Baseline Leukocyte Mitochondrial DNA Copy Number with High-Sensitivity C-Reactive Protein Trajectory After Excluding Participants with High-Sensitivity C-Reactive Protein >3 mg/L During Follow-Up. Supplementary Table 4. Association between Estimated High-Sensitivity C-Reactive Protein Trajectory and Risk of Mortality. Supplementary Table 5. E-Values for the Association of Features of Estimated High-Sensitivity C-Reactive Protein Trajectory Over Time With Risk of Mortality in All Participants. Supplementary Table 6. E-Values for the Association of Features of Estimated High-Sensitivity C-Reactive Protein Trajectory Over Time With Risk of Mortality after the Exclusion of Participants With High-Sensitivity C-Reactive Protein > 10 mg/L During Follow-Up. Supplementary Table 7. E-Values for the Association of Features of Estimated High-Sensitivity C-Reactive Protein Trajectory Over Time With Risk of Mortality after the Exclusion of Participants With High-Sensitivity C-Reactive Protein > 3 mg/L During Follow-Up. [file 12979_2022_322_MOESM1_ESM.docx]

**supplementary MATERIALS**

**Supplementary Figure 1.** The assumed relationship among leukocyte mitochondrial DNA copy number, serum high-sensitive C-reactive protein, risk of death

**Supplementary Methods** Measurement of Covariate Variables, Statistical Analyses, References

**Supplementary Table 1.** Characteristics of HALST Participants Excluded from This Study

**Supplementary Table 2.** Association of Baseline Leukocyte Mitochondrial DNA Copy Number with High-Sensitivity C-Reactive Protein Trajectory After Excluding Participants with High-Sensitivity C-Reactive Protein >10 mg/L During Follow-Up

**Supplementary Table 3.** Association of Baseline Leukocyte Mitochondrial DNA Copy Number with High-Sensitivity C-Reactive Protein Trajectory After Excluding Participants with High-Sensitivity C-Reactive Protein >3 mg/L During Follow-Up

**Supplementary Table 4.** Association between Estimated High-Sensitivity C-Reactive Protein Trajectory and Risk of Mortality

**Supplementary Table 5.** E-Values for the Association of Features of Estimated High-Sensitivity C-Reactive Protein Trajectory Over Time With Risk of Mortality in All Participants

**Supplementary Table 6.** E-Values for the Association of Features of Estimated High-Sensitivity C-Reactive Protein Trajectory Over Time With Risk of Mortality after the Exclusion of Participants With High-Sensitivity C-Reactive Protein > 10 mg/L During Follow-Up

**Supplementary Table 7**. E-Values for the Association of Features of Estimated High-Sensitivity C-Reactive Protein Trajectory Over Time With Risk of Mortality after the Exclusion of Participants With High-Sensitivity C-Reactive Protein > 3 mg/L During Follow-Up

**Supplementary Figure 1.** The assumed relationship among leukocyte mitochondrial DNA copy number, serum high-sensitive C-reactive protein, risk of death.

The figure displayed the overall hypothesized relationships among leukocyte mitochondrial DNA copy number, longitudinal hs-CRP levels, and risk of death that this study aimed to test.**
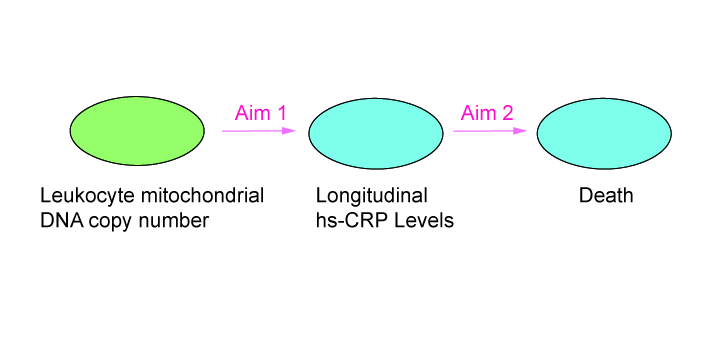
**

**Supplementary Methods**

**Measurement of Covariate Variables**

BMI was defined as measured body weight in kilograms divided by the square of measured height in square meters. Hypertension was defined on the basis of each individual’s self-report doctor’s diagnosis, medication use, or an average measured blood pressure of ≥140/90 mmHg [1-3]. Diabetes mellitus was defined on the basis of each individual’s self-report doctor’s diagnosis, medication use, fasting blood glucose of 126 mg/dL or higher or hemoglobin A1c of ≥6.5% [2-4]. Chronic kidney disease was defined as an estimated glomerular filtration rate of <60 mL·min^−1^ (1.73 m^2^) ^−1^ [5]. Other diseases were defined on the basis of each individual’s self-report doctor’s diagnosis [2]. Serum glucose, creatinine, triglycerides, HDL cholesterol, and LDL cholesterol were determined enzymatically using the ADVIA^®^ 1800 Chemistry System (Siemens AG, Munich, Germany), with coefficients of variation of 1.13%, 3.13%, 1.78%, 2.00%, and 1.52%, respectively.

**Statistical Analyses**

Based on the framework of the random effects joint model, the joint longitudinal and survival modeling applied in this study allows jointly analyzing two common types of outcomes in longitudinal studies, namely longitudinal and time-to-event outcomes [6-9]. A major feature of the joint longitudinal and survival modeling was the capacity of this modeling to properly account for the interdependency between the repeatedly measured longitudinal data process and the event time data process in a longitudinal study, leading to unbiased estimates. On the one hand, the modeling enables the analysis of longitudinal data (repeated measured hs-CRP) with informative censoring owing to the event's occurrence (death); on the other, unlike the traditional Cox model with a time-varying covariate, the modeling provides an accurate (unbiased) estimation of the associations between the endogenous time-varying covariate (hs-CRP) with biological variations and survival. As such, this single method offers a unique opportunity to unbiasedly and simultaneously estimate the associations of baseline exposure/treatment (leukocytes mtDNA_CN_) with the longitudinal outcome (longitudinal hs-CRP levels) (Aim 1) and the association of the longitudinal outcome (longitudinal hs-CRP levels) with the time-to-event outcome (mortality risk) (Aim 2).

The joint longitudinal and survival modeling in this study consisted of a longitudinal part (Equation 1) and a survival part (Equation 2). Let *y_ij_* represents the measured hs-CRP levels of the *i*th participant at time *t_ij_*, Y*_i_*(*t_ij_*) represents the true hs-CRP levels of the *i*th participant at time *t_ij._*, m*_i_* represents the leukocytes mtDNA_CN_ of the *i*th participant, and *z_ki_* denotes the vector of covariates k (taking the values of the *i*th participant). Then

*y_ij_* = Y*_i_*(*t_ij_*) +*e_ij_* (1)

Y*_i_*(*t_ij_*) = *β_0_* + *β_0m_* m*_i_* + *β_0k_z_ki_* + *β_1_ t_ij_* + *β_1m_* m*_i_* *t_ij_* + *β_1k_z_ki_ t_ij_* + *u_0i_* + *u_1i_ t_ij_*

where *e_ij_* represents the measurement error, *u_0i_* represents a participant-specific random intercept, and *u_1i_* represents a participant-specific random slope.

The survival part of the joint modeling is linked to the longitudinal part through Y*_i_*(*t_ij_*). Let *h*(t|x) represents the hazard for death at time *t* for an observation with a predictor taking the value *x*. Then, on the basis of the current value parameterization,

*h_i_* (t| Y*_i_*(t), *z_ki_*) = *h_0_*(t) exp[*α*Y*_i_*(t) + *θ_k_z_ki_*] (2-1)

where *h_0_*(t) is the baseline hazard function and *α* denotes the parameter estimate of the association between the instantaneous hs-CRP level at time t and the risk of death.

If we instead assume a cumulative effects parameterization, the survival part becomes

*h_i_* (t| Y*_i_*(t), *z_ki_*) = *h_0_*(t) exp[γ$\int_{\text{0}}^{\text{t}} \text{Y}_{\text{i}}\text{(s) ds}$ + *θ_k_z_ki_*] (2-2)

where γ denotes the parameter estimate of the association between the area under the hs-CRP trajectory curve up to time *t* and the risk of death.

This joint longitudinal and survival modeling enabled us to test mainly two effects, namely: (1) the effect of the leukocytes mtDNA_CN_ on the longitudinal serum hs-CRP levels (*β_0m_* + *β_1m_ t*), (2) the effect of the longitudinal serum hs-CRP levels on the time to death (*α* for the instantaneous hs-CRP level and γ for the area under the hs-CRP trajectory curve).

References

1. Chobanian AV, Bakris GL, Black HR, Cushman WC, Green LA, Izzo JL, Jr., et al. Seventh report of the Joint National Committee on Prevention, Detection, Evaluation, and Treatment of High Blood Pressure. Hypertension. 2003; 42(6):1206-1252.

2. Wu IC, Lin CC, Liu CS, Hsu CC, Chen CY, Hsiung CA. Interrelations Between Mitochondrial DNA Copy Number and Inflammation in Older Adults. J Gerontol A Biol Sci Med Sci. 2017; 72(7):937-944.

3. Wu IC, Hsu CC, Chen CY, Chuang SC, Cheng CW, Hsieh WS, et al. Paradoxical Relationship Between Glycated Hemoglobin and Longitudinal Change in Physical Functioning in Older Adults: A Prospective Cohort Study. J Gerontol A Biol Sci Med Sci. 2019; 74(6):949-956.

4. American Diabetes Association. Standards of medical care in diabetes--2014. Diabetes Care. 2014; 37 Suppl 1:S14-80.

5. Levey AS, Stevens LA, Schmid CH, Zhang YL, Castro AF, 3rd, Feldman HI, et al. A new equation to estimate glomerular filtration rate. Ann Intern Med. 2009; 150(9):604-612.

6. Rizopoulos D. Joint Models for Longitudinal and Time-to-Event Data: With Applications in R, 1st edn. Boca Raton, FL: Chapman and Hall/CRC; 2012.

7. Ibrahim JG, Chu H, Chen LM. Basic concepts and methods for joint models of longitudinal and survival data. J Clin Oncol. 2010; 28(16):2796-2801.

8. Asar Ö, Ritchie J, Kalra PA, Diggle PJ. Joint modelling of repeated measurement and time-to-event data: an introductory tutorial. Int J Epidemiol. 2015; 44(1):334-344.

9. Wulfsohn MS, Tsiatis AA. A joint model for survival and longitudinal data measured with error. Biometrics. 1997; 53(1):330-339.

**Supplementary Table 1. Characteristics of HALST Participants Excluded from This Study** ^a^

|  | **Excluded** | **Included** |  |
| --- | --- | --- | --- |
| **Characteristics** | (n = 1,735) | (n = 3,928) | ***P*** ^b^ |
| High-sensitivity C-reactive protein, median (interquartile range), mg/L | 4.19 (2.80-7.55) | 0.39 (0.16-0.83) | <.001 |
| Age, years | 70.5 (8.4) | 69.1 (8.1) | <.001 |
| Women, n (%) | 928 (53.5) | 2060 (52.4) | 0.469 |
| Education |  |  |  |
| Illiteracy, n (%) | 248 (14.3) | 370 (9.4) | <.001 |
| Elementary school, n (%) | 837 (48.2) | 1667 (42.4) |  |
| Junior high school, n (%) | 206 (11.9) | 447 (11.4) |  |
| ≥ High school, n (%) | 442 (25.5) | 1444 (36.8) |  |
| Smoking |  |  |  |
| Never, n (%) | 1171 (67.5) | 2830 (72.1) | <.001 |
| Former smoker, n (%) | 305 (17.6) | 634 (16.1) |  |
| Current smoker, n (%) | 259 (14.9) | 464 (11.8) |  |
| Hypertension, n (%) | 1028 (59.3) | 1988 (50.6) | <.001 |
| Diabetes mellitus, n (%) | 556 (32.1) | 1021 (26.0) | <.001 |
| Cardiovascular disease, n (%) | 416 (24.0) | 799 (20.3) | .002 |
| Stroke, n (%) | 115 (6.6) | 188 (4.8) | .005 |
| Lung disease, n (%) | 74 (4.3) | 116 (3.0) | .013 |
| Chronic kidney disease, n (%) | 314 (18.1) | 559 (14.2) | <.001 |

a Data are mean (standard deviations) unless otherwise specified.

b Continuous variables were analyzed using a one-way analysis of variance, whereas categorical variables (proportions) were analyzed using the chi-square test

**Supplementary Table 2. Association of Baseline Leukocyte Mitochondrial DNA Copy Number with High-Sensitivity C-Reactive Protein Trajectory After Excluding Participants with High-Sensitivity C-Reactive Protein >10 mg/L During Follow-Up ^a^**

| **Model** |  | **Model 1** |  | |  | **Model 2** |  |
| --- | --- | --- | --- | --- | --- | --- | --- |
| **Variable** | **Effects** ^b^ | **95% CI** | | ***P*** | **Effects** ^b^ | **95% CI** | ***P*** |
| Intercept | 0.00 | -0.04 to 0.03 | | .788 | -2.55 | -2.99 to -2.11 | <.001 |
| Mitochondrial DNA copy number | -0.06 | -0.09 to -0.03 | | <.001 | -0.05 | -0.07 to -0.02 | .002 |
| Age |  |  | |  | 0.01 | 0.01 to 0.02 | <.001 |
| Sex |  |  | |  |  |  |  |
| Women |  |  | |  |  | (Reference) |  |
| Men |  |  | |  | -0.24 | -0.32 to -0.17 | <.001 |
| Smoking |  |  | |  |  |  |  |
| Never |  |  | |  |  | (Reference) |  |
| Former smoker |  |  | |  | 0.15 | 0.06 to 0.25 | .002 |
| Current smoker |  |  | |  | 0.27 | 0.17 to 0.37 | <.001 |
| BMI |  |  | |  | 0.06 | 0.05 to 0.07 | <.001 |
| Serum triglycerides |  |  | |  | 0.001 | 0.001 to 0.002 | <.001 |
| Serum high-density lipoprotein cholesterol |  |  | |  | -0.005 | -0.007 to -0.002 | <.001 |
| Serum low-density lipoprotein cholesterol |  |  | |  | 0.004 | 0.003 to 0.005 | <.001 |
| Hypertension |  |  | |  | 0.02 | -0.04 to 0.08 | .514 |
| Chronic kidney disease |  |  | |  | 0.10 | 0.01 to 0.19 | .038 |

a The results of the longitudinal part of the joint analysis, in which the association between the baseline leukocyte mitochondrial DNA copy number and the serum high-sensitivity C-reactive protein levels (both of which were natural log-transformed and standardized) was modeled using linear mixed regression with adjustment for other model variables.

b Change in high-sensitivity C-reactive protein levels (in 1 standard deviation).

**Supplementary Table 2. Association of Baseline Leukocyte Mitochondrial DNA Copy Number with High-Sensitivity C-Reactive Protein Trajectory After Excluding Participants with High-Sensitivity C-Reactive Protein >10 mg/L During Follow-Up (continued) ^a^**

| **Model** |  | **Model 1** |  | |  | **Model 2** |  |
| --- | --- | --- | --- | --- | --- | --- | --- |
| **Variable** | **Effects** ^b^ | **95% CI** | | ***P*** | **Effects** ^b^ | **95% CI** | ***P*** |
| Time | 0.06 | 0.05 to 0.07 | | <.001 | 0.03 | 0.01 to 0.04 | .001 |
| Mitochondrial DNA copy number x time | 0.00 | 0.00 to 0.01 | | .87 | Not included |  |  |
| Sex x time |  |  | |  |  |  |  |
| Women |  |  | |  |  | (Reference) |  |
| Men |  |  | |  | 0.02 | 0.00 to 0.04 | .018 |
| Education x time |  |  | |  |  |  |  |
| Illiteracy |  |  | |  | 0.07 | 0.03 to 0.10 | <.001 |
| Elementary school |  |  | |  | 0.03 | 0.01 to 0.05 | .001 |
| Junior high school |  |  | |  | 0.02 | 0.00 to 0.05 | .088 |
| ≥ High school |  |  | |  |  | (Reference) |  |
| Chronic kidney disease x time |  |  | |  | 0.04 | 0.01 to 0.07 | .006 |

a The results of the longitudinal part of the joint analysis, in which the association between the baseline leukocyte mitochondrial DNA copy number and the serum high-sensitivity C-reactive protein levels (both of which were natural log-transformed and standardized) was modeled using linear mixed regression with adjustment for other model variables.

b Change in high-sensitivity C-reactive protein levels (in 1 standard deviation).

**Supplementary Table 3. Association of Baseline Leukocyte Mitochondrial DNA Copy Number with High-Sensitivity C-Reactive Protein Trajectory After Excluding Participants with High-Sensitivity C-Reactive Protein >3 mg/L During Follow-Up ^a^**

| **Model** |  | **Model 1** |  | |  | **Model 2** |  |
| --- | --- | --- | --- | --- | --- | --- | --- |
| **Variable** | **Effects** ^b^ | **95% CI** | | ***P*** | **Effects** ^b^ | **95% CI** | ***P*** |
| Intercept | -0.04 | -0.08 to -0.01 | | .009 | -2.64 | -3.08 to -2.20 | <.001 |
| Mitochondrial DNA copy number | -0.05 | -0.09 to -0.02 | | .001 | -0.04 | -0.07 to -0.01 | .009 |
| Age |  |  | |  | 0.01 | 0.01 to 0.02 | <.001 |
| Sex |  |  | |  |  |  |  |
| Women |  |  | |  |  | (Reference) |  |
| Men |  |  | |  | -0.23 | -0.31 to -0.15 | <.001 |
| Smoking |  |  | |  |  |  |  |
| Never |  |  | |  |  | (Reference) |  |
| Former smoker |  |  | |  | 0.15 | 0.05 to 0.25 | .002 |
| Current smoker |  |  | |  | 0.28 | 0.17 to 0.38 | <.001 |
| BMI |  |  | |  | 0.06 | 0.05 to 0.07 | <.001 |
| Serum triglycerides |  |  | |  | 0.001 | 0.001 to 0.002 | <.001 |
| Serum high-density lipoprotein cholesterol |  |  | |  | -0.004 | -0.006 to -0.001 | .003 |
| Serum low-density lipoprotein cholesterol |  |  | |  | 0.003 | 0.003 to 0.004 | <.001 |
| Hypertension |  |  | |  | 0.02 | -0.04 to 0.08 | .496 |
| Chronic kidney disease |  |  | |  | 0.08 | -0.01 to 0.18 | .077 |

a The results of the longitudinal part of the joint analysis, in which the association between the baseline leukocyte mitochondrial DNA copy number and the serum high-sensitivity C-reactive protein levels (both of which were natural log-transformed and standardized) was modeled using linear mixed regression with adjustment for other model variables.

b Change in high-sensitivity C-reactive protein levels (in 1 standard deviation).

**Supplementary Table 3. Association of Baseline Leukocyte Mitochondrial DNA Copy Number with High-Sensitivity C-Reactive Protein Trajectory After Excluding Participants with High-Sensitivity C-Reactive Protein >3 mg/L During Follow-Up (continued) ^a^**

| **Model** |  |  | **Model 1** |  | |  | **Model 2** |  |
| --- | --- | --- | --- | --- | --- | --- | --- | --- |
| **Variable** | **Effects** ^b^ |  | **95% CI** | | ***P*** | **Effects** ^b^ | **95% CI** | ***P*** |
| Time | 0.02 |  | 0.01 to 0.03 | | <.001 | 0.01 | -0.01 to 0.02 | .361 |
| Mitochondrial DNA copy number x time | 0.00 |  | 0.00 to 0.01 | | .236 | Not included |  |  |
| Sex x time |  |  |  | |  |  |  |  |
| Women |  |  |  | |  |  | (Reference) |  |
| Men |  |  |  | |  | 0.01 | -0.01 to 0.02 | .99 |
| Education x time |  |  |  | |  |  |  |  |
| Illiteracy |  |  |  | |  | 0.05 | 0.02 to 0.08 | .001 |
| Elementary school |  |  |  | |  | 0.02 | 0.00 to 0.04 | .018 |
| Junior high school |  |  |  | |  | 0.01 | -0.01 to 0.04 | .258 |
| ≥ High school |  |  |  | |  |  | (Reference) |  |
| Chronic kidney disease x time |  |  |  | |  | 0.01 | -0.01 to 0.04 | .408 |

a The results of the longitudinal part of the joint analysis, in which the association between the baseline leukocyte mitochondrial DNA copy number and the serum high-sensitivity C-reactive protein levels (both of which were natural log-transformed and standardized) was modeled using linear mixed regression with adjustment for other model variables.

b Change in high-sensitivity C-reactive protein levels (in 1 standard deviation).

**Supplementary Table 4. Association between Estimated High-Sensitivity C-Reactive Protein Trajectory and Risk of Mortality ^a^**

|  | **All** | | | **Excluding** **participants (n=82) with hs-CRP >10 mg/L during follow-up** | | | **Excluding participants (n= 319) with hs-CRP >3 mg/L during follow-up** | | |
| --- | --- | --- | --- | --- | --- | --- | --- | --- | --- |
| **Trajectory feature** | **Hazard ratio** ^b^ | **95% CI** | ***P*** | **Hazard ratio** ^b^ | **95% CI** | ***P*** | **Hazard ratio** ^b^ | **95% CI** | ***P*** |
| Area under hs-CRP trajectory curve (mg/L × years) |  |  |  |  |  |  |  |  |  |
| Unadjusted | 1.05 | 1.04 to 1.07 | <.001 | 1.05 | 1.03 to 1.07 | <.001 | 1.04 | 1.01 to 1.07 | <.001 |
| Multivariable adjusted ^c^ | 1.03 | 1.02 to 1.04 | <.001 | 1.03 | 1.02 to 1.04 | <.001 | 1.02 | 1.01 to 1.04 | <.001 |

a Hs-CRP, high-sensitivity C-reactive protein.

b Hazard ratio per 1 standard deviation increase in hs-CRP levels × years.

c Adjusted for age, sex, educational level, smoking status, obesity (BMI), dyslipidemia (serum triglycerides, HDL cholesterol, LDL cholesterol), hypertension, cardiometabolic diseases (diabetes mellitus, stroke, cardiovascular disease), lung disease, and chronic kidney disease.

**Supplementary Table 5.** **E-Values for the Association of Features of Estimated High-Sensitivity C-Reactive Protein Trajectory Over Time With Risk of Mortality in All Participants ^a^**

| **Trajectory features** | **Hazard ratio ^b^** | **95% CI** | **E-value** | **95% CI** |
| --- | --- | --- | --- | --- |
| Instantaneous hs-CRP level |  |  |  |  |
| Unadjusted | 1.36 | 1.27 to 1.46 | 1.79 | 1.64 |
| Multivariable adjusted ^c^ | 1.22 | 1.14 to 1.30 | 1.56 | 1.42 |
| Area under hs-CRP trajectory curve |  |  |  |  |
| Unadjusted | 1.05 | 1.04 to 1.07 | 1.23 | 1.19 |
| Multivariable adjusted ^c^ | 1.03 | 1.02 to 1.04 | 1.17 | 1.14 |

a Hs-CRP, high-sensitivity C-reactive protein.

b Hazard ratio per 1 standard deviation increase in hs-CRP levels.

c Adjusted for age, sex, educational level, smoking status, obesity (BMI), dyslipidemia (serum triglycerides, HDL cholesterol, LDL cholesterol), hypertension, cardiometabolic diseases (diabetes mellitus, stroke, cardiovascular disease), lung disease, and chronic kidney disease.

**Supplementary Table 6. E-Values for the Association of Features of Estimated High-Sensitivity C-Reactive Protein Trajectory Over Time With Risk of Mortality after the Exclusion of Participants With High-Sensitivity C-Reactive Protein > 10 mg/L During Follow-Up ^a^**

| **Trajectory features** | **Hazard ratio** ^b^ | **95% CI** | **E-value** | **95% CI** |
| --- | --- | --- | --- | --- |
| Instantaneous hs-CRP level |  |  |  |  |
| Unadjusted | 1.37 | 1.25 to 1.48 | 1.79 | 1.60 |
| Multivariable adjusted ^c^ | 1.21 | 1.11 to 1.31 | 1.54 | 1.37 |
| Area under hs-CRP trajectory curve |  |  |  |  |
| Unadjusted | 1.05 | 1.03 to 1.07 | 1.23 | 1.18 |
| Multivariable adjusted ^c^ | 1.03 | 1.02 to 1.04 | 1.16 | 1.12 |

a Hs-CRP, high-sensitivity C-reactive protein.

b Hazard ratio per 1 standard deviation increase in hs-CRP levels.

c Adjusted for age, sex, educational level, smoking status, obesity (BMI), dyslipidemia (serum triglycerides, HDL cholesterol, LDL cholesterol), hypertension, cardiometabolic diseases (diabetes mellitus, stroke, cardiovascular disease), lung disease, and chronic kidney disease.

**Supplementary Table 7. E-Values for the Association of Features of Estimated High-Sensitivity C-Reactive Protein Trajectory Over Time With Risk of Mortality after the Exclusion of Participants With High-Sensitivity C-Reactive Protein > 3 mg/L During Follow-Up ^a^**

| **Trajectory features** | **Hazard ratio** ^b^ | **95% CI** | **E-value** | **95% CI** |
| --- | --- | --- | --- | --- |
| Instantaneous hs-CRP level |  |  |  |  |
| Unadjusted | 1.37 | 1.20 to 1.54 | 1.80 | 1.52 |
| Multivariable adjusted ^c^ | 1.19 | 1.06 to 1.31 | 1.50 | 1.25 |
| Area under hs-CRP trajectory curve |  |  |  |  |
| Unadjusted | 1.04 | 1.01 to 1.07 | 1.19 | 1.08 |
| Multivariable adjusted ^c^ | 1.02 | 1.01 to 1.04 | 1.15 | 1.10 |

a Hs-CRP, high-sensitivity C-reactive protein.

b Hazard ratio per 1 standard deviation increase in hs-CRP levels.

c Adjusted for age, sex, educational level, smoking status, obesity (BMI), dyslipidemia (serum triglycerides, HDL cholesterol, LDL cholesterol), hypertension, cardiometabolic diseases (diabetes mellitus, stroke, cardiovascular disease), lung disease, and chronic kidney disease.
